# Targeting thymidine phosphorylase as a potential therapy for bone loss associated with periprosthetic osteolysis

**DOI:** 10.1002/btm2.10232

**Published:** 2021-06-08

**Authors:** Gen Matsumae, Tomohiro Shimizu, Yuan Tian, Daisuke Takahashi, Taku Ebata, Hend Alhasan, Shunichi Yokota, Ken Kadoya, Mohamad Alaa Terkawi, Norimasa Iwasaki

**Affiliations:** ^1^ Department of Orthopedic Surgery, Faculty of Medicine and Graduate School of Medicine Hokkaido University Sapporo Japan; ^2^ Global Institution for Collaborative Research and Education (GI‐CoRE), Frontier Research Center for Advanced Material and Life Science Bldg No 2. Hokkaido University Sapporo Japan

**Keywords:** aseptic loosening, kinase inhibitor, macrophage factors, TYMP

## Abstract

Macrophages are generally thought to play a key role in the pathogenesis of aseptic loosening through initiating periprosthetic inflammation and pathological bone resorption. The aim of this study was to identify macrophage‐derived factors that promote osteoclast differentiation and periprosthetic bone destruction. To achieve this, we examined the effects of 12 macrophage‐derived factors that were identified by RNA‐seq analysis of stimulated macrophages on osteoclast differentiation. Surprisingly, thymidine phosphorylase (TYMP) was found to trigger significant number of osteoclasts that exhibited resorbing activities on dentine slices. Functionally, TYMP knockdown reduced the number of osteoclasts in macrophages that had been stimulated with polyethylene debris. TYMP were detected in serum and synovial tissues of patients that had been diagnosed with aseptic loosening. Moreover, the administration of TYMP onto calvariae of mice induced pathological bone resorption that was accompanied by an excessive infiltration of inflammatory cells and osteoclasts. The RNA‐seq for TYMP‐induced‐osteoclasts was then performed in an effort to understand action mode of TYMP. TYMP stimulation appeared to activate the tyrosine kinase FYN signaling associated with osteoclast formation. Oral administration of saracatinib, a FYN kinase inhibitor, significantly suppressed formation of bone osteolytic lesions in a polyethylene debris‐induced osteolysis model. Our findings highlight a novel molecular target for therapeutic intervention in periprosthetic osteolysis.

## 1 INTRODUCTION

Total joint arthroplasty (TJA) is an excellent and the most appropriate approach for the treatment of end‐stage arthritic diseases in that it allows joint function to be preserved and also provides pain relief. Implant design and fixation, surgical procedures, infections, and aseptic loosening are the main cases of TJA failure. However, aseptic loosening, a process that is referred to as periprosthetic osteolysis, is the most frequent cause of TJA failure with incidence rates estimated to be 10–70%. It is estimated that over 25% of all TJAs fail due to aseptic loosening, a condition that requires revision surgery.^1^ Osteolysis mainly occurs due to inflammatory response initiated by wear particles that are released from sliding surface of prosthetic materials. Persistent local inflammatory response promotes a bone remodeling process typified by increased osteoclast activities and bone loss that leads to the loss of prosthesis fixation and implant failure.^2^, ^3^, ^4^ Neither anti‐inflammatory nor anti‐resorptive agents have proven to prevent the progression of osteolysis or prolong the lifespan of an implant. Effective therapy is needed to prevent osteolysis as a step toward improving the quality of life for patients and reducing economic burden due to expenses of health service.

Ultra‐high‐molecular‐weight‐polyethylene (UHMWPE) remains the gold standard for prosthetic bearing materials with substantial resistance to abrasion and wear. However, despite the strength of UHMWPE, wear continues to be the major determinant of the lifetime of a prosthesis.^2^, ^3^ Indeed, wear debris that is deposited in the periprosthetic space with rates exceeding 0.15 mm/year have been documented with a high risk of occurrence of periprosthetic osteolysis.^2^ UHMWPE particles with sizes of 0.1 and 10 μm has been frequently been detected in periprosthetic tissues, and particles with sizes of 0.1–2.0 μm are thought to be the most biologically active trigger of high inflammatory responses to pathological bone resorption.^4^

With respect to implants, macrophages are the first line of cells that interact with implanted medical devices. Wear particles are recognized and phagocytosed by resident macrophages in periprosthetic tissues and bone that produce an array of proinflammatory mediators that facilitate cell recruitment, maturation, proliferation, and differentiation. In addition to their function in inflammation, macrophages, including those that are resident in periprosthetic tissues, bone and bone marrow play prominent roles in bone remodeling through producing osteoclastogenic factors and their ability to differentiate into bone resorbing osteoclasts.^5^, ^6^ Indeed, it is known that macrophage‐derived cytokines including TNF‐α, IL‐1, IL‐6, and TNFSF15 promote the differentiation of osteoclast, either directly or indirectly, by inducing the expression of the receptor activator of nuclear factor kappa B ligand (RANKL), a key regulator of osteoclastogenesis, in lymphocytes, osteoblasts and fibroblasts.^6^, ^7^, ^8^, ^9^, ^10^ Consistent with this view, our recent study demonstrated that macrophages stimulated with UHMWPE debris express gene signatures similar to those for rheumatoid arthritis including molecules associated with inflammatory response and osteoclast differentiation.^10^ Specifically, inflammation and pathological bone resorption are the primary features of the pathogenesis of periprosthetic osteolysis and rheumatoid arthritis.^11^ In both diseases, macrophages play a principal role in the development of inflammatory lesions and joint erosion.^11^, ^12^, ^13^ Therefore, studying crosstalk between inflammatory macrophages that are resident in periprosthetic tissues and osteoclast progenitors around an implant may provide clues to the development of effective medical therapies for the treatment of periprosthetic osteolysis.

Given the importance of molecular mediators of macrophages that are stimulated by wear particles as attractive therapeutic targets for preventing periprosthetic osteolysis, the objective of this study was to identify macrophage‐derived factors that are directly involved in osteoclast differentiation. Our study identified a novel potential osteoclastogenic factor and highlighted a promising molecular target for therapeutic interventions in implant loosening.

## 2 RESULTS

### 2.1 TYMP is macrophage‐derived factor inducing osteoclast differentiation

**FIGURE 1:**
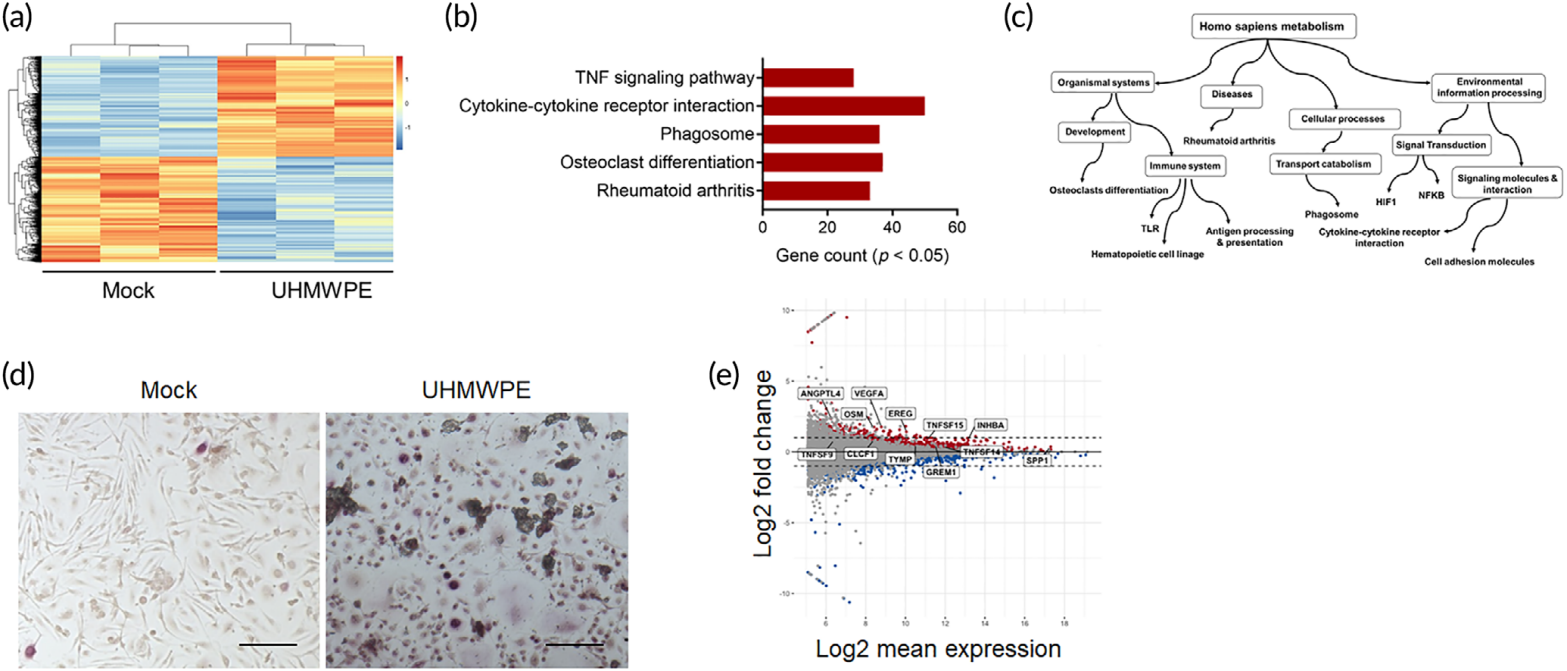
Stimulation macrophages by UHMWPE particles induced osteoclast differentiation. (a) Heat map of regulated genes in macrophages that were stimulated with UHMWPE particles for 24 h (*n* = 3). Log2 fold change >1.0. A scale bar for intense color change from −1 or below indicated by blue color and 1 or above indicated by red. (b) KEGG pathways enrichment analysis of upregulated genes in response to UHMWPE particles stimulation. (c) Acyclic graph for NET‐work‐based gene enrichment of the enriched KEGG pathways (*p* ≤ 1e−07). (d) Representative images for TRAP‐stained macrophages stimulated with UHMWPE particles. Arrows indicate osteoclasts. Scale bars represent 100 μm. (e) MA plot analysis for transcript expression levels of significantly up‐ or downregulated genes in stimulated macrophages. Selected targeted molecules are indicated

**FIGURE 2:**
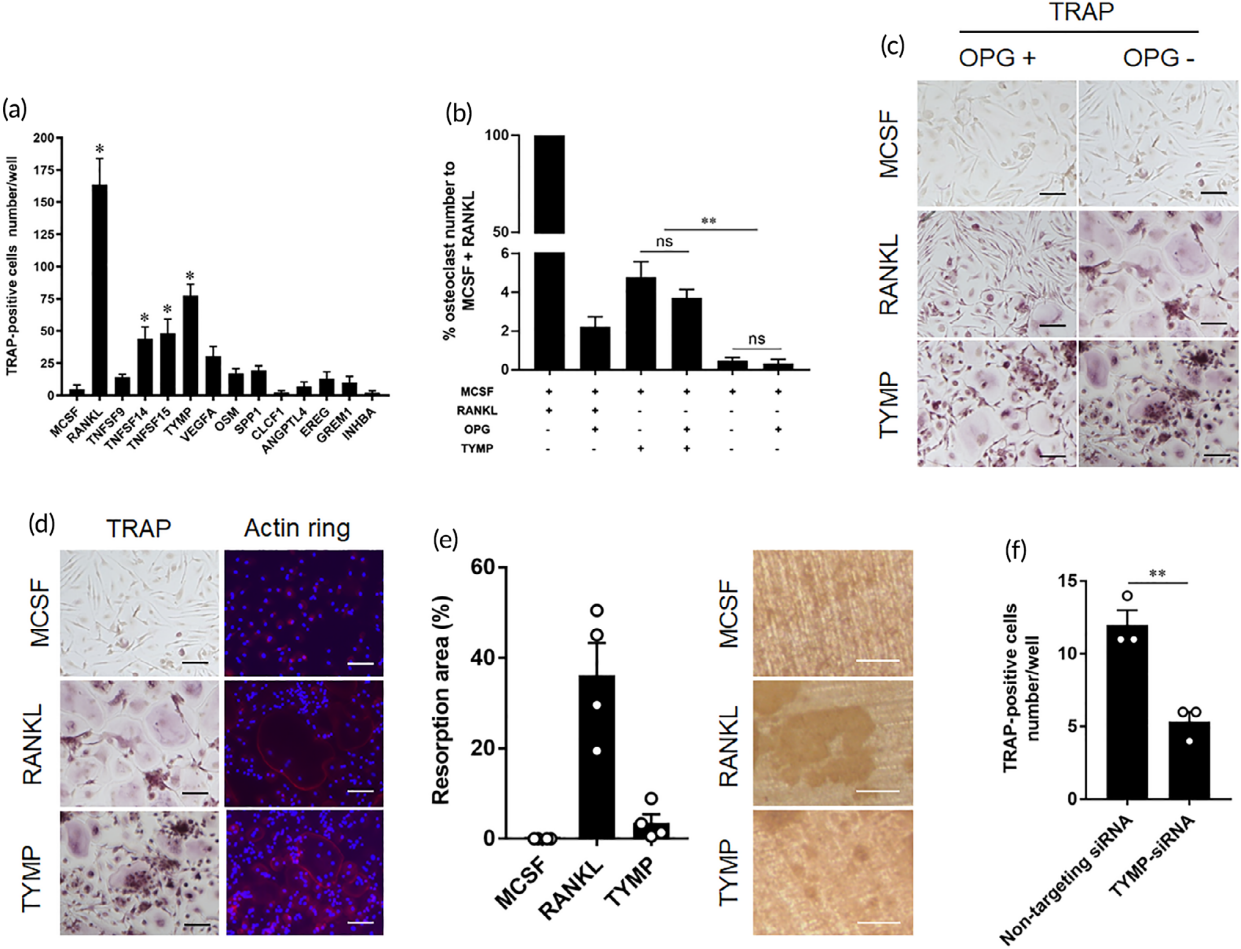
Identification of TYMP as osteoclastogenic factor. (a) Effects of macrophage derived factors on osteoclasts differentiation. Human monocytes were stimulated with each recombinant protein for 8 days and then stained by TRAP for osteoclast detection. The results represent the means ± SEM for triplicates and * indicates a significant difference of cell count as compared to control. (b) Inhibitory effects of OPG on osteoclast differentiation. Human monocytes were stimulated with RANKL or TYMP plus inhibitory dose of OPG. The results represent the means ± SEM for triplicates and significant differences were determined based on one‐way ANOVA, followed by the Tukey's multiple‐comparison procedure. **p < 0.01. No significant differences were indicated as ns. (c) Representative images for TRAP‐stained cells stimulated with RANKL or TYMP in presence or absence of OPG. Scale bars represent 100 μm. (d) Representative images for osteoclasts induced by RANKL or TYMP. Left panel, cells stained by TRAP, and right panel for cells fluorescently stained by phalloidin for detection of Actin ring formation. Actin ring (red), and cell nuclei (blue). Scale bars represent 100 μm. (e) Quantitative analysis of bone resorption area on dentine slices (*n* = 4). The right figure shows the bone resorption area on a dentine slice. Scale bars represent 50 μm. (f) Effects of TYMP knockdown on osteoclast differentiation in macrophages stimulated with UHMWPE particles. Results represent the means ± SEM for triplicates and significant differences were determined Student's *t*‐test. **p < 0.01

To identify macrophage‐secreted factors that are involved in development of pathological bone resorption associated with periprosthetic osteolysis, we first analyzed gene profiles of macrophages that had been stimulated with UHMWPE particles. In the case of macrophages that had been stimulated by UHMWPE particles for 24 h, 2143 genes that were most significantly enriched in osteoclast differentiation based on an analysis of the KEGG pathway were upregulated (Figure 1a,b). To assess the potential functional associations between the genes and biological cellular processes, a NETwork‐based Gene Enrichment was performed for the significantly upregulation genes. The findings revealed that the stimulated macrophages expressed the gene signature of rheumatoid arthritis, including genes that are involved in inflammatory responses and osteoclast differentiation (Figure 1c). To confirm this finding, we cultured human macrophages for 6 days in the presence or absence of UHMWPE particles. Matured osteoclasts were clearly observed in cell cultures that had been stimulated by UHMWPE particles (Figure 1d), suggesting that stimulation with wear particles induced macrophages to differentiate into osteoclasts. Based on these results, we speculated that wear particle‐stimulated macrophages secreted factors that could potentially promote osteoclast differentiation. Of inflammatory‐related molecules, 12 target molecules that were found to be highly expressed in rheumatoid arthritis were selected and their ability to induce osteoclast differentiation in vitro was then examined (Figure 1e). Osteoclast differentiation assays showed that thymidine phosphorylase (TYMP) had the highest osteoclastogenic potential, as evidenced by the increased number of TRAP‐positive cells (Figure 2a). The number of TRAP‐positive cells was not significantly reduced in the TYMP‐stimulated cells after treatment with recombinant osteoprotegerin (OPG) at a concentration of 500 ng/mL (Figure 2b,c). In addition, multinucleated osteoclasts that were induced by TYMP stimulation formed actin rings similar to that induced by RANKL stimulation and exhibited considerable resorbing activity on dentine slices (Figure 2d,e). To gain further insights into the osteoclastogenic function of TYMP, a functional gene knockout experiment was performed in macrophages that had been stimulated with UHMWPE particles. Interestingly, TYMP knockdown reduced the number of osteoclasts in stimulated macrophages, underlining its potential role in osteoclast formation associated with periprosthetic osteolysis (Figure 2f). These collective results suggest that TYMP is a potential osteoclastogenic factor that is released from macrophages stimulated with UHMWPE particles.

### 2.2 TYMP is present in periprosthetic tissues around loosening implants

**FIGURE 3:**
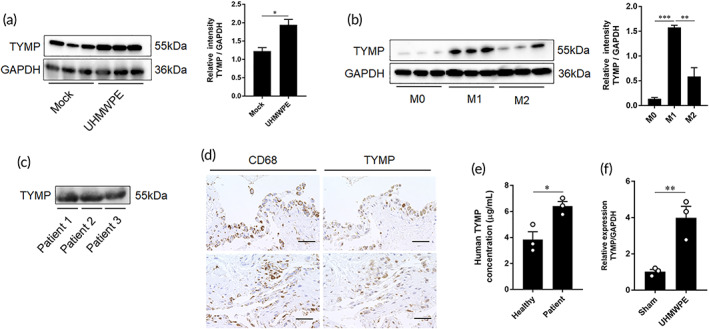
Detection of TYMP in stimulated macrophages and periprosthetic tissues. (a) Western blot analysis of stimulated macrophages by UHMWPE particles. Right panel for quantitative analysis of band intensities. Significant differences indicated as *p < 0.05. (b) Expression of TYMP in M0, M1, and M2 macrophages. Right panel for quantitative analysis of band intensities. Significant differences indicated as **p < 0.01, and ***p < 0.001. (c) Detection of TYMP in synovial fluids collected from patients diagnosed with aseptic loosening of hip arthroplasty by Western blot analysis. (d) Representative images for immunohistochemistry staining of synovial tissues from aseptic loosening patient. Signals for CD68 and TYMP in tissues. Scale bars are 50 μm. (e) Detection of TYMP in the sera of aseptic loosening patients determined by ELISA. Results represent the means ± SEM for three samples and significant difference was determined Student's *t*‐test in comparison to values detected in sera of healthy donors (*n* = 3). (f) Expression of TYMP in calvarial bone tissues after implantation of UHMWPE particles as analyzed by qRT‐PCR. Results represent the means ± SEM for three samples and significant difference was determined by Student's *t*‐test. *p < 0.05, **p < 0.01

To verify whether TYMP is, in fact, the osteoclastogenic factor derived from inflammatory macrophages and is involved in periprosthetic osteolysis, the expression of TYMP was examined in stimulated macrophages, and periprosthetic tissues and sera of patients diagnosed with aseptic loosening. The protein level of TYMP was significantly increased in UHMWPE particles that had been stimulated and M1 polarized macrophages (Figure 3a,b). Likewise, high levels of TYMP were detected in synovial fluid from patients who were undergoing revision surgery (Figure 3c). Consistent with these results, positive signals representing TYMP overlapping CD68+ cells were observed in periprosthetic tissues of the same patients (Figure 3d). On the other hand, only a weak reaction of TYMP was observed in the synovial tissue that was undergoing primary TJA (Supplementary Figure 1). TYMP levels in the sera of patients that were diagnosed with aseptic loosening were significantly elevated compared with those in healthy donors (Figure 3e). To confirm our finding that TYMP is upregulated in response to wear debris, a murine osteolysis model was used and the expression of TYMP was examined in calvarial bone after implanting UHMWPE particles. These mice showed at least a 4‐fold increase in TYMP expression in calvarial bone tissues as compared to those of sham mice (Figure 3f). These results suggest the potential involvement of TYMP in the development of periprosthetic osteolysis.

### 2.3 TYMP triggers local bone erosion in a murine calvarial osteolysis model

**FIGURE 4:**
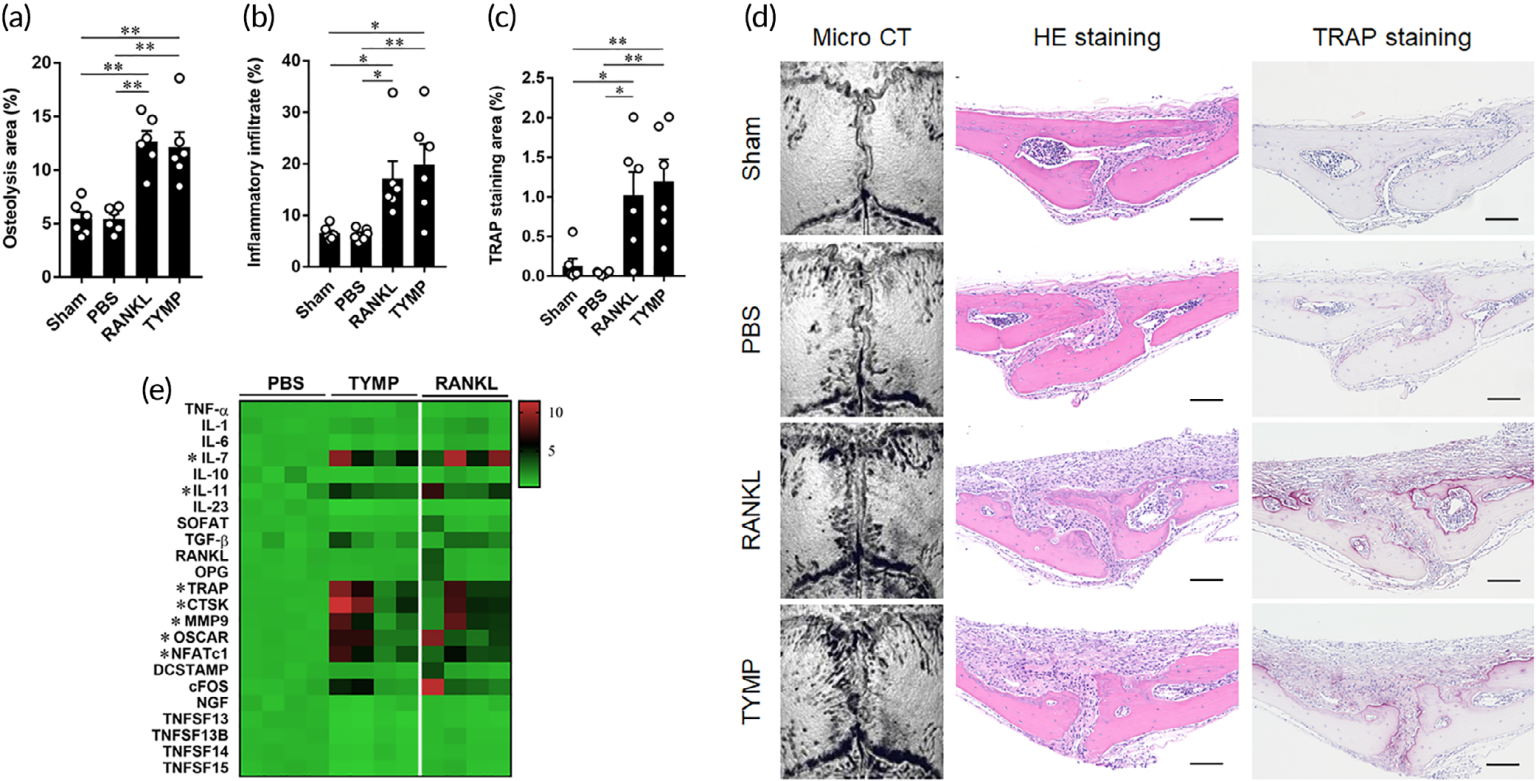
Osteolytic lesions triggered by administration of TYMP on calvarial bone tissues. (a) Quantitative analysis of osteolytic area on the calvarial bone tissues of mice as determined by micro‐CT. (b) Quantification of inflammatory infiltrates in calvarial bone sections. (c) Quantitative analysis of the TRAP staining area in the same tissues. Results represent the means ± SEM for six samples and significant differences were determined by one‐way ANOVA, followed by Tukey's multiple‐comparison procedure. *p < 0.05, **p < 0.01. (d) Representative images for micro‐CT and histological analyses of bone sections stained by HE and TRAP. Scale bars are 100 μm. Red indicates osteoclast stained area in TRAP‐stained sections. (e) Gene expression of inflammatory‐ and osteoclast‐associated molecules analyzed in calvarial bone. Results are visualized heat map representing relative expression values of each target gene after normalizing to the expression of GAPDH ± SEM of four mice. * indicates a significant difference, as determined by the one‐way ANOVA, followed by Tukey's multiple‐comparison procedure

To collect additional evidence on the osteolytic function of TYMP, we performed in vivo experiments using a murine calvarial osteolysis model. Sponges that were soaked in TYMP were transplanted onto the calvariae and lesions histomorphometric analysis was carried out on day 7 and a gene expression analysis was carried out on day 4. Bone assessment by micro‐CT showed that TYMP triggered the development of osteolytic lesions that were comparable to those induced by RANKL and were significantly larger than those of controls (Figure 4a). Histologically, these mice had significantly greater inflammatory infiltrates and TRAP‐stained areas than those in sham mice and mice that had been treated with PBS‐soaked sponges (Figure 4b–d). Consistent with these results, the transplantation of TYMP‐soaked sponges stimulated the production of inflammatory molecules and osteoclast associated factors, including IL‐7, IL‐11, TRAP, CTSK, MMP9, OSCAR, and NFATc1 (Figure 4e). From these results, we conclude that TYMP can directly trigger the local bone erosions typified by the increased production of inflammatory cells and osteoclast infiltration.

### 2.4 TYMP activates tyrosine kinase FYN signaling associated with osteoclasts formation

**FIGURE 5:**
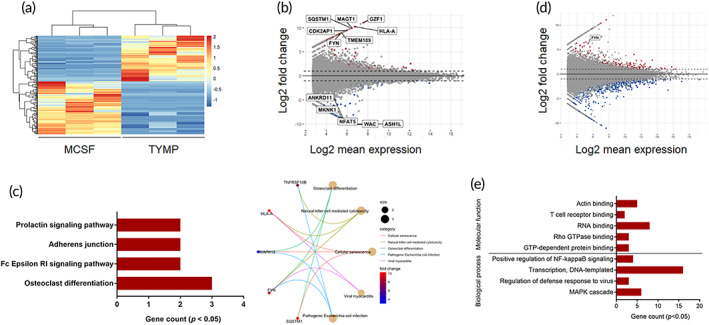
Gene profile of TYMP‐induced osteoclasts. (a) Heat map for the standardized transcript expression levels in human monocytes stimulated with TYMP for 8 days. Control cells were cultured in same medium containing MCSF alone (*n* = 3). Hierarchical clustering for all the transcripts was performed using the longest‐range method. (b) MA plot analysis for transcript expression levels of significantly up‐ or downregulated genes (log2 fold change >2.0; *p* < 0.05) in response to TYMP stimulation (TYMP vs. MCSF). Top‐regulated genes are indicated. (c) KEGG pathways enrichment analysis of upregulated genes. Right panel for circle plot showing the relationship between the significant terms and endogenous factors in the KEGG pathway. (d) MA plot analysis for transcript expression levels of significantly up‐ or downregulated genes (log2 fold change >2.0; *p* < 0.05) in response to TYMP stimulation (TYMP vs. RANKL). (e) Gene Ontology enrichment analysis for top‐enriched terms in molecular function and biological process

**FIGURE 6:**
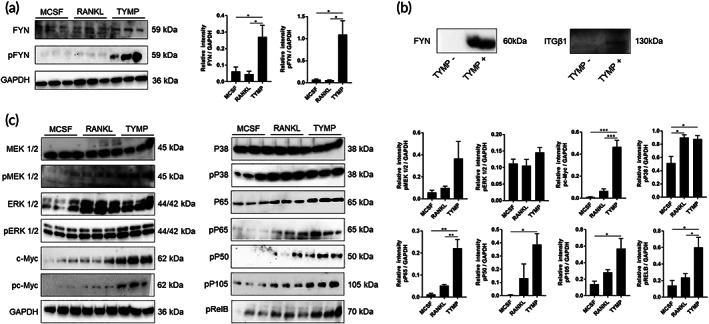
Characterizations of macrophages response to TYMP stimulation. (a) Detection of total and phosphorylated FYN in stimulated human monocytes with TYMP or RANKL for 3 h using Western blot analysis. Right panel shows quantitative analysis of band intensities. (b) Detection of FYN and ITGβ1 in complex proteins pulled down by recombinant TYMP. TYMP‐bound His60 Ni beads were incubated with human monocytes lysate protein and then texted by Western blot analysis. (c) Detection of MAPKs‐ and NF‐κB‐associated molecules in TYMP‐induced osteoclasts. Results represent means ± SEM for triplicates and significant differences were determined by the one‐way ANOVA, followed by Tukey's multiple‐comparison procedure. *p < 0.05, **p < 0.01, and ***p < 0.001

To understand the mechanism by which TYMP induced osteoclast differentiation, we performed a comprehensive gene expression analysis using RNA sequencing for TYMP‐induced osteoclasts. The bioinformatic analysis revealed that, 94 genes that were significantly upregulated and were mainly enriched in osteoclast differentiation term based on KEGG pathway enrichment analysis (Figure 5a–c). The osteoclast differentiation markers included FYN (FC: 8.88), SQSTM1 (FC: 9.92), and MAPK12 (FC: 3.00). Interestingly, expression of FYN was significantly elevated in TYMP‐induced osteoclasts as compared to these induced by RANKL (Figure 5d). Likewise, the top gene ontology terms included positive regulators of osteoclast differentiation such as the MAPK cascade and the regulation of NF‐κB signaling for biological processes and actin binding for molecular function (Figure 5e). Giving the importance of FYN in activating the MAPK and NF‐κB pathways, we next studied the association between TYMP stimulation and FYN expression. It should be noted here that the levels of total and phosphorylated FYN were significantly elevated after a 3 h stimulation with TYMP (Figure 6a). Likewise, stimulated macrophages exhibited an elevation in the gene expression of FYN, as evidenced by quantitative real‐time polymerase chain reaction (qRT‐PCR) (Supplementary Figure 2). More interestingly, a pull‐down assay showed that TYMP was also able to bind a complex protein that contained ITGβ1 and FYN (Figure 6b), suggesting a close association between TYMP and FYN signaling. To further verify our finding that TYMP stimulation increased the expression of MAPK and NF‐κB, which are important transcription factors for osteoclast differentiation, we examined the levels of expression of P38, ERK1/2, MEK1/2, c‐Myc, P65, NFKB1, RELB in macrophages that had been stimulated with TYMP for 6 days. Remarkably, expressions of phosphorylated c‐Myc, P38, P65, NFKB1, RELB were significantly elevated compared to control (Figure 6c). Expression of phosphorylated c‐Myc, P65, and RELB were significantly higher than these in cells that were stimulated with RANKL (Figure 6c). Moreover, there was a tendency toward significance for the increased expression of phospho‐MEK1/2 and phospho‐ERK1/2 in cell stimulated with TYMP (Figure 6c). These results revealed that TYMP activates integrin‐FYN signaling resulting in the initiation of the MAPK and NF‐κB signaling pathways that promote osteoclasts differentiation. These collective findings suggest that TYMP‐FYN axis signaling in osteoclast differentiation is a promising target for preventing pathological bone resorption in periprosthetic osteolysis.

### 2.5 Therapeutic effects of FYN inhibition in murine osteolysis model

**FIGURE 7:**
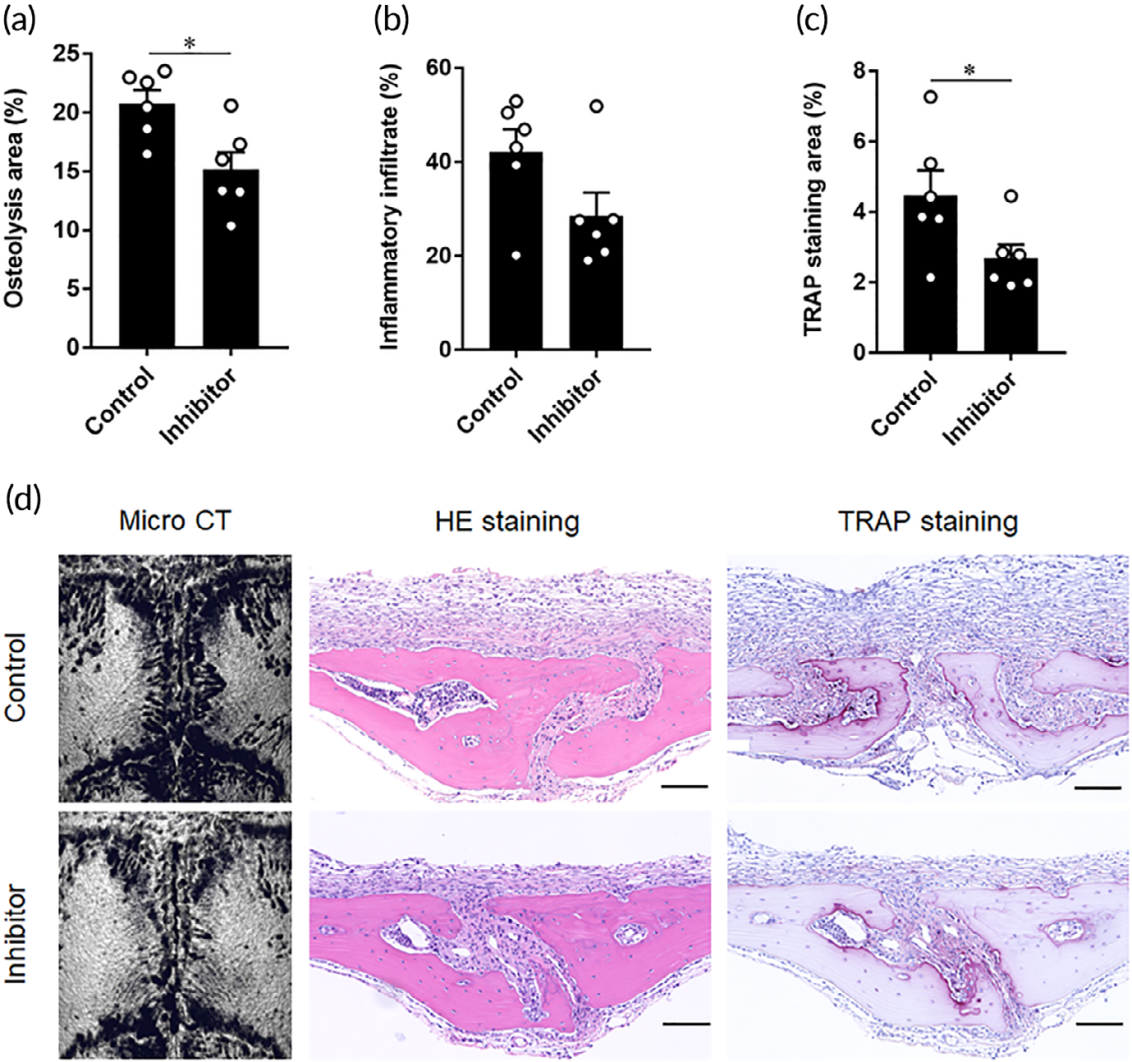
Therapeutic effects of oral administration of saracatinib in UHMWPE particles induced‐osteolysis murine model. (a) Quantitative analysis of osteolytic areas based on micro‐CT images. (b) Quantitative analysis of inflammatory infiltrates in sections of calvarial bone tissues. (c) Quantitative analysis of the TRAP‐stained area in sections of calvarial bone tissues. Mice received inhibitor solvent are control and these received saracatinib are inhibitor group. Results represent means ± SEM for six samples and * indicates a significant difference, as determined by the Student's *t*‐test. *p < 0.05. (d) Representative images for micro‐CT images, HE‐stained images, and TRAP‐stained images in each group. Red indicates osteoclast stained area in TRAP‐stained sections. Scale bars are 100 μm

To test our hypothesis that FYN is a promising molecular target for therapy of periprosthetic osteolysis, saracatinib, a Src kinase inhibitor, was used to inhibit FYN function in a UHMWPE particle‐induced osteolysis model. UHMWPE particles were implanted into calvarial bone and saracatinib was orally administered on days −1, 1, 3, and 5. Bone lesions were evaluated by micro‐CT and histology on day 7. Micro‐CT bone assessment showed a significant reduction in osteolytic regions in the saracatinib‐treated mice as compared to the control (Figure 7a). Consistent with these results, this treatment suppressed inflammatory infiltration and reduced the sizes of TRAP‐stained regions (Figure 7b–d). Taken together, our results shed light on a possible new molecular target for the treatment of periprosthetic osteolysis.

## 3 DISCUSSION

Several lines of evidence suggest that immune factors that are released from inflammatory macrophages act directly on osteoclast progenitors, promoting differentiation into mature osteoclasts and consequently increase bone resorption. In periprosthetic osteolysis, the deposition of UHMWPE particles in periprosthetic tissues leads to the prolonged activation of resident macrophages that produce factors promoting osteoclastogenesis and bone loss around the implant. Macrophages are heterogeneous immune cells that are present in all tissues and are polarized into the proinflammatory M1 and anti‐inflammatory M2 phenotypes. A unique population of bone‐resident macrophages has been recently identified as osteomacs, and are expected to play essential roles in the bone remodeling process due to their being located adjacent to osteoblasts in the endosteal surface of bone. Both inflammatory macrophages and bone‐resident macrophages are expected to produce an array of cytokines in response to microenvironmental stimuli.^11^, ^14^ These proinflammatory factors initiate the remodeling of synovium and bone tissues around the joint and implant leading to focal bone resorption and osteolysis. Indeed, antibodies to these molecules have been clinically approved for the treatment of rheumatoid arthritis, a disease that is closely related to periprosthetic osteolysis, namely, anti‐TNF‐α drugs (infliximab, adalimumab, certolizumab, and golimumab)^15^ and anti‐IL‐6 drug (Tocilizumab).^16^ Moreover, several studies have suggested that blocking cytokines could be a potential therapeutic strategy for periprosthetic osteolysis with promising effects in experimental models.^17^, ^18^, ^19^ In the current study, we identified TYMP as a novel osteoclastogenic factor associated with the development of bone loss in periprosthetic osteolysis.

Our results showed that TYMP was most potent osteoclastogenic factor among the molecules that were tested, including molecules that were earlier reported to induce osteoclast differentiation namely TNFSF14, TNFSF15, and VEGFA.^10^, ^20^, ^21^ TYMP appeared to induce osteoclast production in presence of OPG, a RANKL receptor that functions as its inhibitor and the resulting osteoclast phenotype exhibited substantial bone resorption activities. TYMP was further detected in macrophages that had infiltrated into periprosthetic tissues in patients who were undergoing revision surgery for hip arthroplasty. Interestingly, the knockdown of TYMP in macrophages resulted in a significant reduction in the number of osteoclasts in UHMWPE particle‐stimulated macrophages cultures. TYMP triggered the formation of lytic bone lesions that were comparable to these induced by RANKL in mouse osteolysis models. These collective data suggest that TYMP is one of the osteoclastogenic factors associated with development of periprosthetic osteolysis. Consistent with this view, the elevated expression of TYMP has been documented in inflammatory diseases characterized by focal lytic bone lesions, including RA and malignancies of bone, breast, prostate, and lung.^22^, ^23^, ^24^, ^25^ In bone‐metastatic tumors, TYMP binds to integrins in osteoclast progenitors resulting in the activation of PI3K/Akt signaling and an increased methylation of interferon regulatory factor 8 (IRF8), a molecule that enhances the expression of nuclear factor of activated T cells, cytoplasmic 1 (NFATc1), thereby leading to osteoclast differentiation and increased bone resorption.^23^

Generally, TYMP is known as an enzyme that catalyzes the reversible phosphorolysis of thymidine and deoxyuridine. It is mainly expressed in macrophages and plays an important role in angiogenesis and tumor growth, invasion, and metastasis. An increased expression of TYMP is frequently associated with the aggressiveness of cancer with a poor prognosis.^25^, ^26^, ^27^ Intracellular TYMP acts a cytosolic enzyme that is essential for the stability of mitochondrial DNA, and gene mutations cause mitochondrial neurogastrointestinal encephalomyopathy associated with ptosis and progressive external ophthalmoplegia, peripheral neuropathy, severe gastrointestinal dysmotility, cachexia and leukoencephalopathy.^28^ Such facts make targeting this gene difficult due to its essential function in cells. On the other hand, extracellular TYMP secreted from immune cells enhances the expression of proinflammatory mediators, including CXCL10, TNF‐α, IL‐6 and IL‐8, and the production of oxygen species (ROS) through NF‐κB signaling.^22^, ^23^, ^24^, ^25^, ^26^, ^27^, ^29^ Extracellular TYMP activates platelet and thrombosis through binding to SH3 domain‐containing proteins of the Src family kinase (SFK) and increasing integrin activity. In fact, TYMP released from macrophages contributes to the formation of atherosclerotic plaques, thus leading to fatal cardiovascular disorders, including myocardial infarction and stroke.^30^ These findings suggest that TYMP acts as a signaling molecule that participate in the activation of multiple signaling pathways associated with tissue remodeling.

Our further data showed that TYMP stimulation resulted in a significant upregulation of FYN in macrophages in addition to its ability to pull down complex proteins containing FYN and ITGβ1. These results are in agreement with an earlier study showing that TYMP acts as a signaling molecule by directly binding to Src family kinases (SFKs) through its N‐terminus residue, leading to platelet activation and aggregation.^30^ SFKs include Src, Yes, Fyn, Fgr, Lck, Hck, Blk, Lyn, and Frk are a group of non‐receptor tyrosine kinases that are implicated in the survival and function of osteoclasts.^31^ Nonetheless, among the SFKs members, FYN appears to be the most functionally important factor in this process that facilitates the proliferation and differentiation of osteoclasts and retarding their apoptosis.^32^ The engagement of FYN with the integrin receptor family has been documented to be associated with initiating intracellular signaling cascades leading to cell activation, proliferation and differentiation.^30^, ^33^, ^34^ Specifically, an earlier study showed a functional link between ITGβ1 and FYN kinase in mediating the differentiation, maturation and survival of oligodendrocytes.^33^, ^34^ These results highlight the importance of ITGβ1/FYN signaling in driving osteoclast differentiation in response to TYMP stimulation.

Our results suggest a new mechanism that underlines the action of TYMP in osteolysis, because it has been documented that TYMP promotes osteoclastogenesis through downregulating the expression of IRF8, which results in increasing the expression of NFATc1.^23^ Our RNA‐seq data did not show any significant reduction in the expression of IRF8 in TYMP‐stimulated cells, implying a different mode of action. The discrepancy in the results of the two studies can be explained by the different methodologies used in the current study. In fact, we cultured monocytes with TYMP alone, while Luo et al., cultured osteoclast precursors with myeloma cells plus a low dose RANKL.^23^ Generally, osteoclastogenesis is mediated by a canonical pathway (RANKL‐dependent) and a noncanonical pathway. In the canonical pathway, osteoclastogenic effects are dependent on intracellular signaling pathway initiated by the binding of RANKL to its receptor RANK that recruits adaptor proteins TNF receptor‐associated factors (TRAF), mainly TRAF6, which turn on a range of signal transduction pathways, including NF‐κB, MAPKs and AP‐1.^35^ The activation of TRAF6‐NF‐κB and c‐Fos pathways induces the production of NFATc1, a master regulator of osteoclast differentiation, that facilitates cell fusion and osteoclast formation. In the noncanonical pathway, certain cytokines and growth factors, such as TNFα, IL‐6, IL‐11, LIGHT (lymphotoxin exhibiting inducible expression and competing with herpes simplex virus glycoprotein D herpes virus entry mediator, a receptor expressed by T lymphocytes) have been documented to induce osteoclast formation in vitro in the absence of RANKL stimulation.^36^ They can activate signal transduction pathways that are involved in osteoclast formation, including NF‐κB, MAPKs and NFATc1. However, the resulting osteoclasts produced by these factors appear smaller with fewer nuclei and form smaller lacunar resorption pits than those formed in RANKL‐treated cells.^37^ Consistent with these facts, our data clearly indicate that stimulation with TYMP promoted the expression MAPKs and NF‐κB transcription factors in a manner similar to RANKL stimulation. Importantly, an earlier finding suggested the existence of a functional link between FYN and MAPKs and NF‐κB pathways in the activation of T cells.^38^, ^39^ These findings may provide an explanation for the ability of TYMP to function as a signaling molecule participating in multiple signaling pathways.

Regarding the translation of our findings into therapeutics, a clinically approved FYN kinase inhibitor, saracatinib, was used for treating osteolysis induced by UHMWPE particles. Our data demonstrated that the oral administration of saracatinib significantly attenuated the lytic bone lesions in osteolysis murine model. These findings shed light on a promising approach for preventing osteolytic bone lesions in periprosthetic osteolysis. Traditionally, Src inhibitors have proved to have therapeutic advantages for the treatment of osteolytic bones in cancer bone metastasis in mice.^40^ The safety and potential efficacy of saracatinib in suppressing bone resorption might make it a particularly promising agent for the treatment of periprosthetic osteolysis.^41^, ^42^

## 4 MATERIALS AND METHODS

### 4.1 Ethics statement

The institutional review boards of Hokkaido University and hospital approved the study protocols for the use of human samples (approval ID: 016‐0002). All participants signed an informed consent form for research use. Procedures for animal experiments were performed following protocols approved by the Institute of Animal Care and Use Committee of the Hokkaido University Graduate School of Medicine (no. 17‐0085).

### 4.2 Osteoclasts differentiation and bone resorption assays

Human monocytes obtained from the blood of healthy donors (Asian, three males with ages of 30–45 years) were isolated by density gradient centrifugation (Ficoll‐PaqueTM PLUS: GE Healthcare, Waukesha, WI) followed by MACS Pan monocyte isolation kit (Miltenyi Biotec, Auburn, CA).^43^ Cells were suspended in growth medium containing minimum essential medium Eagle (MEM) supplemented with 10% heat‐inactivated fetal bovine serum (FBS), a 5% penicillin/streptomycin solution, and 5% L‐glutamine and cultured in a 37°C‐humidified atmosphere containing 5% CO2 in a 75 cm^2^ flask for 3 h. Thereafter, the medium was discarded and adherent monocytes (CD14^+^) were cultured in growth medium supplemented with 25 ng/mL human recombinant macrophage colony‐stimulating factor (MCSF; Peprotech, Japan). After 3 days of cultivation, the adherent cells were washed 3 times with ice‐cold phosphate‐buffered saline (PBS; Nacalai Tesque, Kyoto, Japan) and detached by treatment with a 1% trypsin–EDTA solution (GE Healthcare). Cells were washed with PBS (Nacalai), seeded on a 96‐well plate (1 × 10^4^) and cultured in growth medium supplemented with 25 ng/mL MCSF (Peprotech, Japan) with or without 50 ng/mL of one growth factors for 8 days. Growth factors included human recombinant proteins of Receptor activator of nuclear factor kappa‐Β ligand (RANKL), TNF superfamily member 9 (TNFSF9), TNF superfamily member 14 (TNFSF14), TNF superfamily member 15 (TNFSF15), Oncostatin M (OSM), osteopontin (SPP1), Epiregulin (EREG) (Peprotech), Vascular endothelial growth factor A (VEGFA; Aviva systems biology, San Diego, CA), Angiopoietin Like 4 (ANGPTL4; Aviscera Bioscience, Inc., CA), TYMP (Aviva systems biology), Gremlin‐1 (GREM1; BioVision, Inc., Milpitas, CA), Cardiotrophin‐like cytokine factor 1 (CLCF1; Aviscera Bioscience, Inc., CA), and Activin A (INHBA; Novoprotein). The growth medium for the cultures was regularly replenished with fresh growth medium supplemented with each recombinant protein at 3‐day intervals. Data from results of triplicate‐well/plate of a single experiment. Cells were stained on day 8 by a Leukocyte acid phosphate TRAP kit (Sigma), and positive cells with ≥3 nuclei were considered to be osteoclasts. Cell count was determined as the number of cells/cm^2^ on 10 random microscopic fields in each well. For cytoskeletal actin staining, cells were fixed using 4% paraformaldehyde (Wako) for 20 min, washed with ice‐cold PBS, and permeabilized using 0.1% triton X100 (Sigma) in PBS for 3 min. Cells were washed by PBS and then stained using an Alexa Fluor 633 phalloidin (Invitrogen, Carlsbad, CA) and DAPI (Dojindo Molecular Technologies) for detection of actin ring formation and nuclei, respectively.^44^ For the bone resorption assay, cells (3 × 10^4^) were cultured on ivory dentine slices (Wako, Osaka, Japan) in growth medium and the recombinant proteins for 21 days. The results represent data from four independent experiments. Dentine slices were stained with a 20 mg/mL solution of peroxidase‐conjugated wheat germ agglutinin (Sigma) and 3,3′‐diaminobenzidine (0.52 mg/mL in PBS containing 0.1% H_2_O_2_). Bone resorption pits on the slices were visualized by confocal microscopy and measured as the percentage of resorbed bone surface per total bone surface area (ImageJ, National Institutes of Health). For the differentiation of macrophages into M1 and M2, monocytes were cultured with 25 ng/mL MCSF for 6 days and then treated for with 100 ng/mL lipopolysaccharide (LPS; Sigma) plus 100 ng/mL recombinant human interferon gamma (IFN‐γ) for M1 macrophages and 200 ng/mL IL‐4 (Peprotech) for M2 macrophages. Cells were harvested after 48 h and lysed for further use.

### 4.3 Immunohistochemistry staining of synovial tissues

Human synovial tissues of patients undergoing revision of total hip arthroplasty due to implant loosening (one male of 60‐years old, two females of 54‐ and 59‐years old) were fixed in 4%‐paraformaldehyde for 48 h and embedded in paraffin. Paraffin blocks were sectioned and sections with 3 μm were incubated with proteinase K (Dako, CA) for 5 min, followed by blocking with horse serum for 1 h. Sections were incubated with 1:250 monoclonal antibody to human TYMP (NeoMarkers, CA) overnight and signals were next amplified with horseradish peroxidase (HRP)‐conjugated streptavidin secondary antibody (Vectastain Elite ABC kit; Vector Laboratories, Burlingame) followed by counterstaining with hematoxylin for detecting cellular nuclei.

### 4.4 Western blotting

Human synovial fluids and cell lysate were mixed samples buffer EzApply (ATTO, Osaka, Japan) and heated at 100°C for 5 min. The extract proteins were subjected to SDS‐PAGE gels and transferred electrophoretically onto polyvinylidene difluoride membrane (Immobilon‐P Membrane; Merck, Darmstadt, Germany). The membranes were blocked in 5% skimmed milk and then incubated with each primary antibody at optimal concentration recommended by manufacturer's instructions. Primary antibodies included those to TYMP (NeoMarkers), FYN (Biolegend), pFYN (R&D Systems, MN), Integrin beta‐1 (ITGβ1; Thermo Fisher Scientific, IL), GAPDH (Affinity Biosciences), P38, pP38, pERK1/2, pMEK1/2, c‐Myc, pc‐Myc, pNFKB1, pRELB (Cell Signaling Technology, MA), and pP65 (Gene Tex, CA). Each respective secondary antibody conjugated with HRP (Cell Signaling Technology) was used for detection of bound antibodies. Signals were detected by Ez WestLumi Plus (ATTO, Tokyo, Japan) and Quantity One v. 4.6.9 (Bio‐Rad) software. Band intensities were quantified based on intensities of target/GAPDH using ImageJ (NIH).

### 4.5 Enzyme‐linked immunosorbent assay

TYMP was detected in human blood sera of three patients and three healthy donors respectively using a quantitative enzyme‐linked immunosorbent assay (ELISA) kit (R&D Systems Inc., MN) according to procedure recommended by the manufacturer's instructions (https://www.rndsystems.com/products/human-pd-ecgf-duoset-elisa_dy229-05).

### 4.6 Preparation of polyethylene particles

Polyethylene particles prepared from hip‐bearing materials of ultra‐high‐molecular‐weight polyethylene as earlier reported.^43^ Briefly, UHMWPE was manufactured from GUR1020 powder (Celanese Japan, Tokyo, Japan) after 95 kGy irradiation and annealed below Tm (135°C) (Quadrant Polypenco Japan, Tokyo, Japan). The solid materials were then crushed by Multi Beads Shocker (Yasui Kikai, Osaka, Japan) at 3500 rpm. Particles with sizes 0.1 to 10 μm were sterilized using an ethylene oxide gas (EOG) sterilizer (Eogelk‐SA‐H160, Osaka, Japan), resuspended in 100 μL PBS and mixed with LAL Reagent of a ToxinSensor Single Test kit (Genscript, Piscataway, NJ) for endotoxin detection. After incubation at 37°C for 1 h in a water bath, a positive reaction was observed according to the manufacturer's instructions by the formation of a firm gel. The formation of a firm gel was not observed in the tested particles indicating that the endotoxin levels were below the detection limit of the kit (0.015 EU/mL). The sterilized free‐endotoxin particles were placed in 1.5 mL Eppendorf tube (6 mg) until use.

### 4.7 Macrophage culture with polyethylene particles and knockdown experiment

Human monocytes were obtained using our routine procedure with purity >98%.^43^ Cells were cultured in medium containing α‐MEM supplemented with 10% heat‐inactivated FBS, 5% penicillin/streptomycin solution, 5% L‐glutamine, 25 ng/mL MCSF (Peprotech) for 6 days. Cells were detached, counted, and seeded 1x10^4^ for knockdown experiment using small interfering RNA (siRNA). Briefly, 25 μL siRNA solution including TYMP and negative (Thermo Fisher, Silencer® Select, Tokyo, Japan) were diluted by Opti‐MEM® (Gibco, Thermo Fisher) and mixed with 25 μL Lipofectamine® RNAiMAX Reagent (Thermo Fisher) diluted by Opti‐MEM®. After 5 min incubation at room temperature, 10 μL siRNA‐lipid complex was added to each well in 96‐well plate. Silencing TYMP was confirmed after 48 h by Western blotting that showed significant reduction of expression (Supplementary Figure 3). After 24 h, the siRNAs‐treated macrophages (1 × 10^4^) were cultured with free‐endotoxin UHMWPE at a density of 1 mg/cm^3^ using the inverted cell culture method^10^ for 24 h. Starting on the 2^nd^ day, cells were cultured using traditional culture methods and the medium was changed on days 1 and 3. Cells were stained by TRAP staining kit (Sigma) after 6 days stimulation and osteoclasts were counted as the number of TRAP^+^cells/cm^2^ on 10 random microscopic fields in each well. Data represent the results of triplicate‐well/plate of a single experiment. All in vitro experiments were repeated at least twice for reproducibility of data.

### 4.8 RNA‐sequence and bioinformatics

Optimal concentration for inducing osteoclast by TYMP treatment was initially determined as 100 ng/mL. Osteoclasts were differentiated by treatment with either RANKL (positive control) or TYMP (at optimal concentration) for 8 days. Monocytes cultured in growth medium supplemented with MCSF (negative control) were washed 3 times by ice‐cold PBS (Nacalai Tesque) and then lysed by TRIzol Reagent (Invitrogen, Thermo Fisher Scientific, CA) for RNA extraction. Total RNA was extracted using RNeasy Plus Mini Kit (Qiagen, Hilden, Germany) according to the manufacturer's instructions, and the integrity of samples was assessed by determining 28S/18S ribosomal RNA bands with an Agilent 2100 bioanalyzer (Agilent Technologies, Santa Clara, CA). High‐quality libraries assayed by Bioanalyzer High sensitivity DNA kit (Agilent) were subjected to NovaSeq 6000 (Illumina). Before the mapping of paired‐end read data, a 3′ terminal base of each read data was trimmed by fastp.^45^ An average of 33 million reads (paired‐end reads of 100 bp) per sample was mapped by alignment to the human genome (GRCh38) using STAR,^46^ gene expression level was quantified using RSEM,^47^ and differentially expressed genes (DEGs) were analyzed using DESeq2 Wald test (R software packages). The significant differences in transcripts were determined by the false discovery rate (FDR) control method with a threshold of p‐value <0.05 by multiple tests. Genes that were expressed at fold change (FC) > 0.0 and FDR < 0.05 compared to negative control (MCSF) were considered significantly differentiated. DEGs were used for Gene Ontology enrichment analysis using clusterProfiler (R software packages). The package was also used to visualize the graphs for the most significantly enriched terms (*p* < 0.05). Network‐based gene enrichment NET‐GE (http://net-ge.biocomp.unibo.it/enrich) was used to visualize acyclic graph for the most significantly enriched terms (*p* ≤ 1e−05). Pathway enrichment analysis was performed using the Database for Annotation Visualization and Integrated Discovery online tools (DAVID: https://david.ncifcrf.gov/). The RNA‐seq data included in this study are publicly available at the Gene Expression Omnibus (GEO) database (https://www.ncbi.nlm.nih.gov/geo/) with an accession number GSE171542.

### 4.9 Quantitative real‐time polymerase chain reaction

Cells and tissues were homogenized and lysed using TRIzol Reagent (Invitrogen) and RNA was extracted using RNeasy Plus Mini kit columns (Qiagen, Hilden, Germany). The cDNAs were synthesized using the GoScriptTM reverse transcriptase kit (Promega, Madison) and assayed using the SYBR® Premix Ex Taq™ II (Takara, Shiga, Japan) and gene‐specific primers listed in supplementary information Table 1 and our earlier study.^44^ Gene expression was determined by the ^2−ΔΔ^Ct method with amplification efficiencies ranging between 90 and 110% for the target and reference genes.

### 4.10 Pull‐down assay

Human monocytes (1.2 × 10^6^) obtained using our routine procedure were lysed by EzRIPA Lysis buffer (Atto, Tokyo, Japan) and protein concentration was determined using Micro BCA™ Protein Assay kit (Thermo Fisher, Tokyo, Japan). Protein lysate was kept in ice until use for pull‐down assay. Thereafter, 100 μL of His60 Ni Magnetic Beads (Takara, Osaka, Japan) was washed by PBS, then was incubated with 10 μg His‐tagged TYMP (ATGEN, Korea) using slow‐speed rotator (AS ONE, Osaka, Japan) for 2 h. The TYMP‐bound beads were next washed by PBST and incubated with 1 mL lysate protein containing 250 μg protein concentration for 2 h. Negative controls included lysate and beads without recombinant TYMP. Protein‐bound‐beads were washed 3 times by PBST and finally subjected to SDS‐PAGE and Western blot analysis. Bound proteins were first tested by Western blot analysis with antibody to irreverent target (GAPDH antibody; Affinity Biosciences) to validate our assay. No reaction was detected with GAPDH antibody (data not shown).

### 4.11 Murine calvarial osteolysis model

For osteolysis model induced by TYMP, 8‐week‐old male C57BL/6 mice (CLEA, Tokyo, Japan) were anesthetized by an intraperitoneal injection of 100 mg/kg ketamine and 10 mg/kg xylazine. Head hair was shaved in preparation for making a sagittal incision (<1 cm) over the calvaria anterior site. Collagen sponges of the single layer type (PELNAC, Tokyo, Japan) were soaked in 4 μg recombinant murine TYMP (Cloud‐Clone Crop., TX) or RANKL (Biolegend) diluted in a total 50 μL PBS and transplanted subcutaneously onto the calvariae for 7 days. Control mice received transplanted sponges that had been soaked in 50 μL PBS. For the UHMWPE particles induced osteolysis model, a sagittal incision (<1 cm) over the calvaria anterior site of C57BL/6 mice was made and 6 mg of UHMWPE particles per mouse were implanted onto the calvarial bone for 7 days. Control mice received the same surgical procedure (Sham) without further particle implantation. The incision was closed using stainless steel clips. Mice were sacrificed and their calvariae were subjected to gene expression analysis by qRT‐PCR (day 4 post implantation) and micro‐CT Analyses (day 7 post implantation) and bone histomorphometry analysis (day 7 post implantation). For the analysis of bone pits, fixed calvariae were analyzed by high‐resolution micro computed tomography (micro‐CT) scanning R_mCT2 scan (Rigaku, Tokyo, Japan) at a 10‐mm isotropic resolution. Pits (%) on the calvariae were calculated by the ImageJ (NIH). The one centimeter square area including the centerline of each calvaria was selected for evaluation as the region of interest. Moreover, 10% formalin‐fixed calvariae were decalcified in 10% EDTA (Wako, Osaka, Japan) for 3 days and then embedded in paraffin. Five‐micron thick sections were stained with hematoxylin and eosin, and tartrate resistance acid phosphatase (TRAP) staining (Sigma‐Aldrich, Missouri) according to the manufacturer's instructions. The one centimeter square, including the centerline of each calvaria was cut and divided into three parts. Two distal regions from the stained sections were analyzed by the ImageJ program (NIH, USA) for the quantification of cell infiltration into tissues.^44^ Each result from the gene expression analysis was performed in quadruplicate and reflected four animals in a single experiment. Each result from the bone histomorphometry analysis was repeated in sextuplicate and reflected six animals in a single experiment. In separate experiment, mice were orally treated with 25 mg/kg saracatinib (Chemscene, NJ) dissolved in dimethyl sulfoxide (DMSO; Wako, Japan) and diluted to 300 μL in corn oil (Wako) on days −1, 1, 3, and 5 post UHMWPE particles implantation. In parallel, control mice were treated by vehicle solution containing DMSO and corn oil (Wako). Calvariae were subjected micro‐CT Analysis and bone histomorphometry analysis on day 7 post implantation. Each result was performed in sextuplicate and reflected six animals in a single experiment.

### 4.12 Statistical analysis

Statistical analyses were performed using GraphPad Software (GraphPad Software Inc., La Jolla, CA). One‐way analysis of variance (ANOVA) followed by Tukey's multiple‐comparison and Student's *t*‐test procedures were used to compare osteoclasts number, percentages of resorbed areas, gene expression, band intensity, bone pits, and cell infiltrates. Results were presented as means ± standard errors of the means (SEM) and were considered statistically significant when *p* < 0.05.

## 5 CONCLUSIONS

In summary, the findings reported here identified TYMP as a potential osteoclastogenic factor that is released from inflammatory macrophages in response to UHMWPE wear stimulation. Our results show a functional association between TYMP stimulation and FYN signaling leading to osteoclast differentiation. FYN therefore has the potential for being a promising therapeutic target for preventing bone loss associated with implant failure in periprosthetic osteolysis.

## CONFLICT OF INTEREST

The authors declare no competing interests.

## AUTHOR CONTRIBUTIONS

**Gen Matsumae:** Conceptualization; data curation; formal analysis; investigation; methodology; validation; visualization; writing‐original draft. **Tomohiro Shimizu:** Funding acquisition; supervision; validation; visualization. **Yuan Tian:** Formal analysis; visualization. **Daisuke Takahashi:** Funding acquisition; methodology; project administration. **Taku Ebata:** Data curation; formal analysis; investigation; methodology. **Hend Alhasan:** Data curation; investigation; methodology. **Shunichi Yokota:** Formal analysis; investigation; visualization. **Ken Kadoya:** Funding acquisition; methodology; project administration; resources. **M Alaa Terkawi:** Conceptualization; data curation; formal analysis; funding acquisition; investigation; methodology; project administration; resources; supervision; validation; writing‐review & editing. **Norimasa Iwasaki:** Conceptualization; project administration; supervision; writing‐review & editing.

### PEER REVIEW

The peer review history for this article is available at https://publons.com/publon/10.1002/btm2.10232.

## Supporting information

**Appendix S1**: Supporting information

## Data Availability

All data needed to evaluate the conclusions in the paper are present in the paper and/or the Supplementary Material. Additional data related to this study might be requested from corresponding author.
