# Supplementary material for: Targeting thymidine phosphorylase as a potential therapy for bone loss associated with periprosthetic osteolysis
Source: Bioeng Transl Med. 2021 Jun 8;6(3):e10232. doi: 10.1002/btm2.10232 (PMC8459589; doi:10.1002/btm2.10232)
Supplement: Supplementary file 1 — Appendix S1: Supporting information [file BTM2-6-e10232-s001.pdf]

Supplementary material

Supplementary Table. Primers used in this study for qRT-PCR.

Target	Forward	Reverse
**hFYN**	TCATGATCTGCGCTTCCTCAA	TGGGCAGTTTGGGGAAGTATG
**mTYMP**	TGCACGCGGTGATAGATGG	AGCATGGCCCCAATTTGTGT
**mTNFα**	AGCCGATGGGTTGTACCTT	ATAGCAAATCGGCTGACG
**mIL-7**	TGCCCGAATAATGAACCAAACT	TGCGAGCAGCACGATTTAGA
**mIL-23**	TAATGTGCCCCGTATCCAGTG	GGCTGTTGTCCTTGAGTCCT
**mCTSK**	CAGAAGCAGTATAACAGCAAGGT	CCCAAATTAAACGCCGAGAGA
**mNGF**	CATCCACCCACCCAGTCTTC	CCAACCCACACACTGACACT
**mTNFSF13**	GCAGTACTCACCCAGAAGCA	TGTTAACTGGAACAAGATGCAGG
**mBAFF**	GTGGTGAGGCAAACAGGCTA	GCAAAGATGGGGTCCGTGTA
**mTNFSF14**	TTGTGGTGGATGGACAGACG	GTCTCCGGTGGTTCTGTTCC
**mTNFSF15**	CACATTCCGAGGGACCACAT	AGTCTGGCTTGTTTGGTCGT

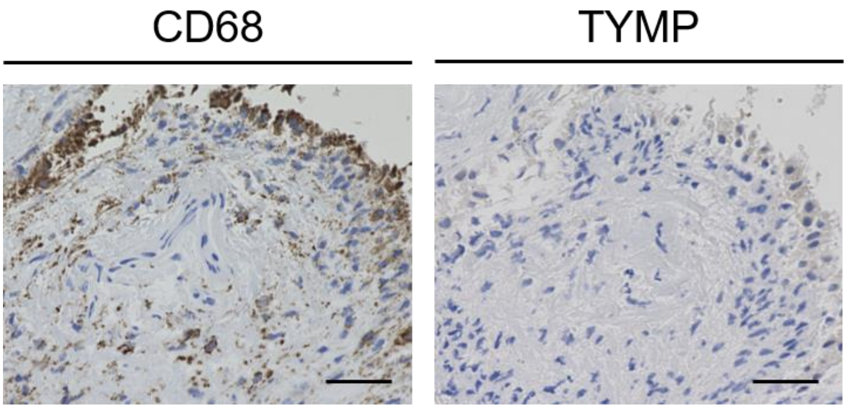

Supplementary FIGURE1 Representative images for immunohistochemistry staining of synovial tissues from primary TJA patient. Formalin-fixed tissues were stained by specific antibody to CD68 and TYMP. Very weak signals for TYMP were detected in tissues. Scale bars are 50 µm.

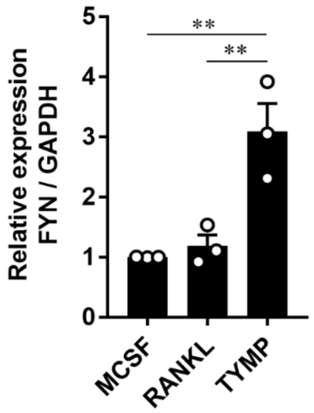

Supplementary FIGURE2 Expression of FYN in macrophages stimulated with cytokines for 3h as analyzed by qRT-PCR. Results represent the means ± SEM for three samples and significant difference was determined by the One-way ANOVA, followed by Tukey’s multiple-comparison procedure.

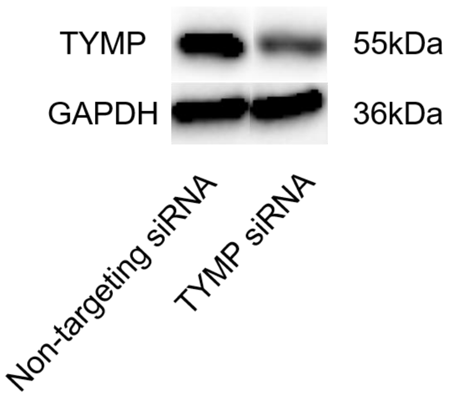

Supplementary FIGURE3 Protein expression of TYMP in macrophages after specific TYMP siRNA treatment. Human macrophages were analyzed by Western blot analysis after 24 h treatment by siRNA for detection of TYMP and GAPDH. Image representative of knockdown of TYMP by siRNA.
